# A practical guide to functional enrichment analysis

**DOI:** 10.3389/fbinf.2026.1895224

**Published:** 2026-08-19

**Authors:** Fábio Henrique Schuster de Oliveira, Lucas Giron dos Santos, Bruno César Feltes

**Affiliations:** Laboratory of DNA Repair and Aging, Department of Biophysics, Institute of Biosciences, Federal University of Rio Grande do Sul, Porto Alegre, Brazil

**Keywords:** benchmark, bioinformatics, functional enrichment, gene ontology, guidelines, overrepresentation analysis

## Abstract

Functional Enrichment Analysis (FEA) has become a critical step in the analysis of omics data in recent years. As a result, numerous approaches have been rapidly developed, leading to an overflow of information that can be difficult for beginners to navigate. Most usage information on FEA methods is scattered across countless articles that often address specific problems, making it difficult to find practical recommendations and good practices. In these guidelines, we discuss the current landscape of FEA and FEA-related approaches and organize actionable knowledge to support first-timers and update veterans on advancements in the field. We aim to aid non-bioinformaticians who use FEA through the general decision-making process necessary to ensure the quality of the analysis. We also provide a practical guide that assists FEA users through the decision-making process underlying FEA and directs readers to more advanced resources for specific FEA contexts. As a basic step in any omics analysis, our practical guide may aid in rapid decision-making for FEA.

## 1 Introduction

Functional enrichment analysis (FEA) has become an almost mandatory step in omics-based studies, serving as a bridge between large-scale gene lists and biological interpretation. Despite such popularity, 75% of papers employing FEA exhibit methodological flaws in their analyses (Wijesooriya et al., 2022). Among the most concerning issues are inadequate background usage and the absence of a statistical correction method, both of which can directly compromise the interpretation of results by altering the set of enriched terms. We hypothesize that this issue stems from actionable usage information being scattered across numerous publications, each of which often addresses only specific aspects of FEA. For instance, a substantial portion of the available literature comprises publications that discuss the use of only a class of methods (Ackermann and Strimmer, 2009; Ihnatova et al., 2018; Ma et al., 2019; Ziemann et al., 2024) and address sole steps or features within FEA (Tomczak et al., 2018; Gable et al., 2022; Ho et al., 2022; Ryan V et al., 2025). Moreover, the information in these specialized studies can be somewhat inaccessible to the average non-bioinformatician FEA user, as it delves into advanced FEA methodological aspects that require prior knowledge beyond that of first-timers, such as the performance difference between univariate and multivariate approaches (Glazko and Emmert-Streib, 2009) or the impact of how the null hypothesis is defined (Ho et al., 2022). Although there are publications that provide comprehensive overviews of FEA, most are outdated given the field’s rapid evolution (Khatri et al., 2012; García-Campos et al., 2015; Mooney and Wilmot, 2015; Maleki et al., 2020; Chicco and Agapito, 2022). Thus, it is of utmost importance to compile such data and translate it into practical guidelines that cover the FEA analysis as a whole while directing users to targeted in-depth sources.

**FIGURE 1:**
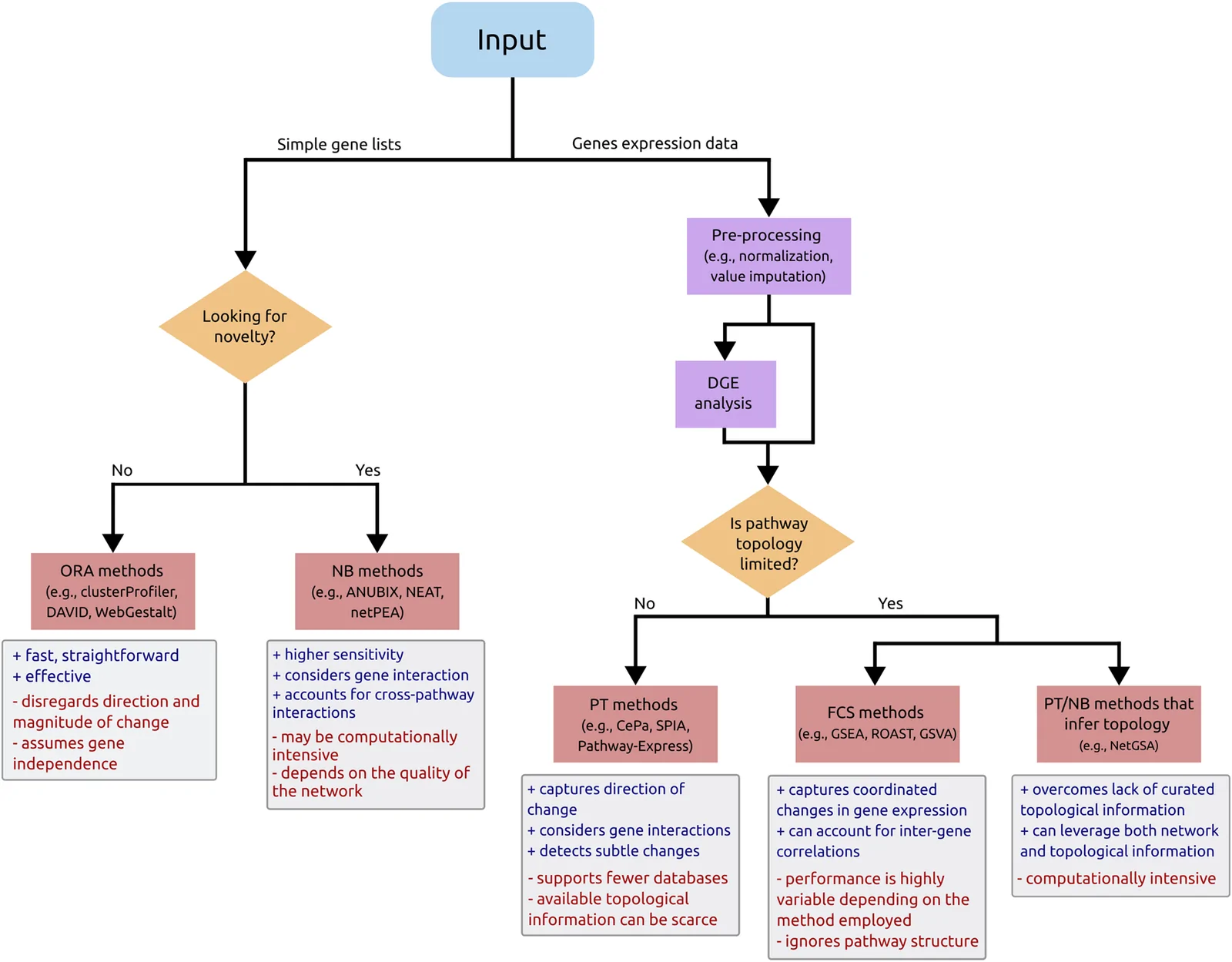
FEA method class decision flowchart.

A plethora of methods have been developed for FEA, each relying on different statistical strategies that can be grouped into 4 distinct classes: Overrepresentation analysis (ORA), Functional Class Scoring (FCS), Pathway-Topology-Based (PT), and Network-Based (NB). Each of these strategies incorporates different levels of biological information into the analysis, ranging from expression values to gene interaction knowledge, and exhibits its own strengths and weaknesses (Khatri et al., 2012; García-Campos et al., 2015). As a consequence, methods from different classes can yield different results that impact the interpretation of the experiment. In this context, performance studies of FEA approaches have become necessary. Most benchmarking efforts in the field have emphasized the ability to detect arbitrarily defined target terms while overlooking the biological informativeness and interpretability of the results (de Oliveira et al., 2026). Because the final stage of FEA inherently relies on manual interpretation, tools should not only balance false discovery rate, but also prioritize biologically relevant descriptions to facilitate meaningful inference. Therefore, metrics such as term specificity, prioritization, and measures of biological novelty provide essential insights that should be the focus of performance assessments.

For newcomers to bioinformatics, evaluating the plethora of available tools can be overwhelming. For seniors, it can be difficult to keep up with new trends in a universe of legacy, well-established tools, where it can be comfortable to use the ones already trusted. In this context, there is a clear need for structured methodological guidelines that help researchers design, execute, and interpret FEA in a biologically rigorous and reproducible manner. The present guidelines propose a general framework to guide users through the multiple decision-making steps during FEA.

We begin by describing the most basic aspects of applying FEA, then delve into the different types of enrichment strategies, their methodological foundations, strengths, limitations, annotation systems, interpretation approaches, and practical recommendations for biologically robust and reproducible analyses. We then create a “guidelines in a nutshell” section and conclude with a discussion of FEA applications. To avoid repetition in the literature, we often redirect readers to other reviews and specialized articles that summarize broader topics not suitable for a guideline.

## 2 Guidelines for FEA

### 2.1 The very basic: input characteristics and research goals’ impact on tool choice

An often-overlooked aspect that researchers should consider when performing FEA is how the biological question itself should guide the selection of the analytical tool. Enrichment platforms are often treated as compatible, meaning that their choice is more a matter of preference or of which one offers greater statistical power than of how they treat biological results. However, our data suggest that different tools implicitly emphasize distinct interpretative strategies, which can substantially shape the biological information extracted from the same dataset.

Some tools tend to prioritize broadly represented and highly annotated biological processes (Klopfenstein et al., 2018; De Oliveira et al., 2026) depending on their methodological strategy and supported databases. While this behavior may appear conservative, it often results in the repeated identification of generic cellular functions, such as metabolism, transcriptional regulation, cell cycle control, and responses to chemical stimuli. For exploratory analyses or studies seeking to capture global biological information, such behavior may be advantageous, as it highlights dominant, well-established biological themes within the dataset. In contrast, other tools exhibit greater stringency or different ranking strategies that tend to emphasize more specific or context-dependent biological processes. These approaches may reduce the prominence of generic terms and instead highlight pathways more closely associated with the specific experimental condition under investigation. For hypothesis-driven studies aiming to uncover new insights or disease-specific pathways, such tools may provide more informative results by prioritizing biologically focused terms. The difference in the presence of broad and specific functional terms in FEA results stems from characteristics of both the functional databases and FEA tools used. For example, Kyoto Encyclopedia of Genes and Genomes (KEGG) contains terms that are, on average, larger (i.e., with more genes annotated to them) than those in Gene Ontology or Reactome. This indicates that KEGG has fewer biologically specific terms and may yield less informative results, since larger terms are more likely to be enriched (Gable et al., 2022). Also, particularities in FEA tools and its methods influence the specificity of the enriched terms retrieved. For instance, topGO (Alexa, 2024) includes multiple algorithms that assess the relationships between related terms and prioritize the most specific ones. Likewise, FEA tools can contain built-in features that eliminate broad descriptions from the analysis or results based on their annotation sizes, such as ClueGO (Bindea et al., 2009) and clusterProfiler (Xu et al., 2024).

Researchers should therefore consider their primary objective before selecting an enrichment platform. In some cases, complementary use of multiple platforms may even provide a more balanced interpretative profile, allowing researchers to distinguish between general biological information and more specialized processes. Hence, enrichment analysis should not be approached as an automated procedure.

### 2.2 Which enrichment tool is right for you?

A crucial step in obtaining insightful results with FEA is mindfully selecting the enrichment method. This decision can significantly influence experimental outcomes and biological interpretation; thus, users should be aware of the method’s strengths and limitations to ensure optimal results. Moreover, it is essential to consider the user’s goals and the nature of the input data in the analysis, as different methods exhibit specific characteristics that make them appropriate for different scenarios. Therefore, the choice of tool should be based primarily on the enrichment methods implemented (Figure 1). Approaches of diverging categories incorporate different types of biological information to calculate enrichment. As a consequence, distinct methods can capture different biological landscapes; thus, combining them helps researchers not only find strong correlations but also retrieve descriptions that might go undetected with a single method (Mooney and Wilmot, 2015; Alhamdoosh et al., 2017). In addition, some tools are developed with a specific scope in mind (e.g., for metabolomics (Zhou and Xia, 2018; Pang et al., 2021), genomics (Sheffield and Bock, 2016; Gu and Hübschmann, 2023), and ncRNA (Olgun et al., 2021; Zhang et al., 2023)), which can be very powerful depending on users’ data and analysis goals. A comprehensive list of these is available in the GSARefDB database (Xie et al., 2021).

As aforementioned, FEA methods can be categorized into four classes–also commonly referred to as generations–according to the underlying enrichment strategy: ORA, FCS, PT, and NB. To help readers select FEA tools, we compiled a list of tools and methods assessed in performance studies that compare at least 4 different approaches (Table 1).

**TABLE 1:** Available FEA tools and methods.

Tool	Platform	Class	Last update	Last database update	Supported functional databases	Custom annotations	FEA visualization	References
WebGestalt	Web/R/Python/Rust	ORA/FCS/NB	2024	2024	10+	Yes	Yes	Elizarraras et al. (2024)
GOstats	R	ORA	2026	2026	GO, Reactome, PFAM	Yes a	No	Falcon and Gentleman (2007)
DAVID	Web	ORA	2026	2025	10	No	Yes	Sherman et al. (2022)
PANTHER	Web	ORA/FCS	2024	2025	GO, Reactome, PANTHERdb	No	No	Mi et al. (2019)
Enrichr	Web	ORA	2025	2025	10+	No	Yes	Chen et al. (2013)
g:Profiler	Web/R	ORA	2023	2026	9	Yes	Yes	Kolberg et al. (2023)
ShinyGO	Web	ORA	2025	2025	10+	No	Yes	Ge et al. (2020)
ClueGO	Cytoscape	ORA	2023	2023 b	7	Yes	Yes	Bindea et al. (2009)
BiNGO	Cytoscape	ORA	2021	2017	GO	Yes	Yes	Maere et al. (2005)
topGO	R	ORA/FCS	2025	2026	GO	Yes	Yes	Alexa (2024)
clusterProfiler	R	ORA/FCS	2026	2026	10+	Yes	Yes	Xu et al. (2024)
goana (limma)	R	ORA	-	2026	GO, KEGG	No	No	
CePa	R	ORA-PT/FCS-PT hybrids	2026	2012	Reactome, KEGG, BioCarta, NCI	Yes	Yes	Gu and Wang (2013)
CERNO (tmod)	R	FCS	-	-	c	Yes	Yes	Zyla et al. (2019)
GSEA	App	FCS	2025	2026	MSigDB (10+)	Yes	Yes	Subramanian et al. (2005)
fGSEA	R	ORA/FCS	2026	-	c	Yes	Yes	Korotkevich et al. (2016)
CAMERA (limma)	R	FCS	-	-	c	Yes	No	Wu and Smyth (2012)
GAGE	R	FCS	2022	2026	GO, KEGG	Yes	Yes	Luo et al. (2009)
GSA	R	FCS	2024	-	c	Yes	Yes	Efron and Tibshirani (2007)
GSVA	R	FCS	2026	-	c	Yes	Yes	Hänzelmann et al. (2013)
PADOG	R	FCS	2026	-	c	Yes	No	Tarca et al. (2012)
ROAST (limma)	R	FCS	-	-	c	Yes	No	Wu et al. (2010)
FRY (limma)	R	FCS	-	-	c	Yes	No	Ritchie et al. (2015)
globaltest	R	FCS	2023	2026	GO, KEGG, MSigDB (10+)	Yes	Yes	Goeman et al. (2004)
SAFE	R	FCS	2026	2026	GO, KEGG, PFAM	Yes	Yes	Barry et al. (2005)
clipper	R	PT	2019	-	graphite (7)	Yes	Yes	Martini et al. (2013)
Pathway-Express (ROntoTools)	R	PT	-	-	KEGG	Yes	Yes	Draghici et al. (2007)
SPIA	R	PT	2025	-	KEGG	Yes	Yes	Tarca et al. (2009)
NetGSA	R	NB-PT hybrid	2026	-	graphite (7)	Yes	Yes	Ma et al. (2016)
DEGraph	R	PT	2026	-	KEGG, NCI	Yes	Yes	Jacob et al. (2012)
PathNet	R	PT	2026	2010	KEGG	Yes a	No	Dutta et al. (2012)
ANUBIX	R	NB	2024	-	c	Yes	No	Castresana-Aguirre and Sonnhammer (2020)
BinoX	Command-line	NB	2017	-	c	Yes	No	Ogris et al. (2017)
NEAT	R	NB	2024	-	c	Yes	Yes	Signorelli et al. (2016)
netPEA	Command-line	NB	2020	-	c	Yes	No	Liu and Ruan (2013)

Table legends: a: no built-in function to input custom annotations; b: contains a built-in function to update databases; c: no specific database support.

All of the tools above have been assessed in performance studies that encompassed at least 5 different tools (Tarca et al., 2013; Bayerlová et al., 2015; Ihnatova et al., 2018; Ma et al., 2019; Maleki et al., 2019; Nguyen et al., 2019; Zyla et al., 2019; Geistlinger et al., 2021; Buzzao et al., 2024; de Oliveira et al., 2026). Deprecated or non-open source methods were discarded.

#### 2.2.1 Overrepresentation analysis (ORA)

ORA is the first-generation method developed for FEA and is also the most straightforward strategy, often requiring the user to simply paste a list of genes and select a few point-and-click parameters. It essentially evaluates whether the overlap between genes of interest (e.g., the user’s DEG list) and a given pathway is statistically significant. The most common ORA methods include Fisher’s exact test, the hypergeometric test, the chi-squared test, the binomial test, and variations of these statistical tests.

It is important to note that ORA methods require a background gene list, which directly influences the magnitude of p-values and the interpretation of the results (Wijesooriya et al., 2022). ORA-based tools frequently use the whole genome as the default background, inflating p-values (lowering them), thereby increasing the risk of false positives. This is particularly critical to ensure biological coherence when the input consists of DEGs, in which case the background should encompass only the genes detected in the experiment, since gene expression varies across tissues. See (Timmons et al., 2015) for an in-depth discussion of this topic. Using the genome as a background may be convenient when a coherent background cannot be easily defined (e.g., when analyzing gene functional clusters in interaction networks or gene lists derived from experiments across different tissues). Regardless, the background used should always be reported by researchers.

As ORA by default only requires simple gene lists, it does not leverage additional biological information, such as gene expression data or gene interaction knowledge. This introduces two major limitations that undermine the biological significance of its results. Firstly, it assumes independence between genes and between pathways. Secondly, ORA requires the user to define an arbitrary threshold for selecting genes of interest from a DEG list, thereby further reducing the biological information. As a consequence, ORA methods tend to provide the most conservative results. Despite these constraints, ORA methods are frequently found among the top performers in FEA performance studies (Xie et al., 2021).

In summary, ORA is suitable for inputs consisting of simple gene lists not derived from experimental data. Moreover, due to its conservative nature and limited ability to detect subtle alterations (Abatangelo et al., 2009; Zyla et al., 2019; Buzzao et al., 2024), ORA might not be adequate when the goal is to identify new, potentially impacted pathways that have not been previously associated with a given context.

#### 2.2.2 Functional class scoring (FCS)

To address the limitations displayed by ORA, FCS incorporates experimental data into the core of its FEA strategy. FCS methods leverage gene-level statistics of differential expression (e.g., fold change, t-statistic, signal-to-noise ratio, and so on) to compute an aggregate pathway-level statistic (e.g., mean, median, sum, Wilcoxon rank sum). This statistic is then compared against the null hypothesis to define enrichment. Because of this framework, FCS tools tend to exhibit higher sensitivity than ORA platforms (Khatri et al., 2012; Zyla et al., 2019). However, this often comes at the cost of longer computational times (Geistlinger et al., 2021; Buzzao et al., 2024).

Although FCS includes a substantial number of parameters that can influence the results, the choice of pathway-level statistic and the definition of the null hypothesis are known to produce the most significant changes in FEA results (Ackermann and Strimmer, 2009). The pathway-level statistics can be classified as univariate, which assumes gene independence within a pathway, or multivariate, which accounts for correlations among genes. Multivariate approaches, such as globaltest (Goeman et al., 2004) or Hotelling’s T2 (Kong et al., 2006), tend to be more sensitive because they can detect subtle, coordinated shifts. Nonetheless, both classes perform similarly on real biological data, though they might yield slightly different sets of enriched pathways. Regarding statistical significance, the null hypothesis is either defined as self-contained or as competitive. The main methodological difference is that, in the self-contained hypothesis, phenotype labels are permuted, whereas in the competitive hypothesis, genes are the units of permutation. The self-contained null hypothesis is generally preferred, as it yields higher statistical power and preserves gene correlations (Subramanian et al., 2005; Ackermann and Strimmer, 2009; Khatri et al., 2012). However, its efficiency depends entirely on the number of samples in the data; thus, when handling fewer samples, the competitive null hypothesis approach is advised (Subramanian et al., 2005; Powers et al., 2018).

It is noteworthy that different FCS tools’ input may require additional preprocessing steps of the expression data. For instance, RNA-seq data should undergo VST normalization for use with microarray-based FEA platforms (Geistlinger et al., 2021). Nonetheless, as a rule of thumb, both RNA-seq and microarray data should be normalized before FEA (Hung et al., 2012; Geistlinger et al., 2021).

#### 2.2.3 Pathway topology-based (PT)

The third generation follows a framework similar to the one employed in FCS. PT methods essentially compute a gene-level statistic that incorporates information regarding the topology of the tested pathways. For instance, instead of simply using expression-derived information, PT methods incorporate metrics that reflect the positions and interactions of genes within a given pathway to define the gene-level statistics. However, it should be emphasized that this is a generalization of the logical strategy underlying PT tools, as the way topological information is incorporated can vary considerably. We direct readers to (Mitrea et al., 2013; Ma et al., 2019) for a thorough assessment of current PT approaches.

As PT methods account for the functional aspects of pathways, they are expected to be more biologically accurate and more likely to detect subtle associations. Because of this, PT tools are especially powerful for investigating phenotypic mechanisms and predicting downstream impacts of gene expression alterations (Nguyen et al., 2019). However, there is still debate about whether increased complexity is advantageous in terms of performance (Bayerlová et al., 2015). Furthermore, the applicability of PT methods depends on the availability of topological information, which can be scarce in some contexts, particularly for non-model organisms. In such cases, tools that predict the pathway topology from expression data, such as NetGSA (Ma et al., 2016), are recommended.

#### 2.2.4 Network-based (NB)

NB tools are often regarded as a part of PT methods. The distinction between the two can sometimes be unclear, since interaction information from biological networks can be considered topological information. However, the key methodological difference is that NB tools primarily rely on network connectivity and crosstalk between gene sets rather than on the topology of the pathways themselves. Most tools in this category leverage connectivity (e.g., the number of links and distances) between the query gene list and the gene sets of the tested pathways to compute enrichment scores, whereas PT methods use pathway-level structural data (often in the form of directed graphs) and rarely consider connectivity between different pathways.

While some tools require gene expression data as input, NB platforms also commonly accept simple gene lists, i.e., without expression-related information, similar to ORA tools. In addition, by exploiting molecular interaction networks, NB tools can account for not only interdependencies among genes but also among pathways, thereby addressing one of the main drawbacks of earlier generations. Recent performance studies have shown that NB and NB-hybrid approaches are among the most sensitive methods, making them particularly well-suited to capturing novelty in biological systems (Dong et al., 2016; Buzzao et al., 2024; Pickholz Berliner and Sharan, 2025). Given this, NB tools are ideal for investigating obscure biological mechanisms within arbitrarily defined gene lists.

Nevertheless, the efficiency of these methods relies, in addition to the algorithm itself, on the quality of the interaction network used. Users should thoughtfully consider the link confidence cutoff and the types of interaction evidence used, and tune these parameters to their goals. For instance, a lower confidence threshold may increase the likelihood of the FEA providing new biological insights, whereas a higher threshold may be more suitable for a more conservative approach.

### 2.3 Where does the information come from? Choosing the annotation system

#### 2.3.1 Functional database enrichment performance

A commonly overlooked step in FEA is selecting the functional database used to retrieve gene annotations, despite its crucial role in the analysis’s success. There are several annotation systems employed in FEA, among them, the Gene Ontology (GO) (Ashburner et al., 2000; Consortium et al., 2023), KEGG pathways (Kanehisa et al., 2000; Kanehisa et al., 2025), Reactome (Milacic et al., 2024), and WikiPathways (Agrawal et al., 2024) are the most commonly embedded in FEA platforms. Each functional database has its own features that can influence the outcome of the enrichment analysis and the biological interpretation of the results. Thus, being aware of database characteristics is critical to ensuring FEA’s success. However, very few articles provide comprehensive analyses of such resources in the context of functional enrichment; thus, only a limited selection of databases has been systematically compared (Gable et al., 2022). Gable et al. assessed the databases’ FEA performance in terms of their novelty-detection potential and the specificity of their results. They found that GO and Reactome, among the most widely used pathway databases, produce the best overall results.

#### 2.3.2 Knowledgebase scope

First and foremost, users should consider which types of information are relevant to their study when selecting the most appropriate functional databases. While the vast majority of FEA tools are centered on pathway gene sets, multiple resources provide different types of annotations tailored to particular contexts, which can be extremely useful depending on the user’s goals. For instance, the GO includes a library that annotates genes to the subcellular locations where they exert their molecular functions. Likewise, the Human Phenotype Ontology (HPO) links gene sets with phenotypic alterations associated with human disease, which can be helpful when investigating disease mechanisms with DGE data. It is worth noting that many FEA tools support multiple functional databases. Therefore, conducting the same analysis using complementary databases can provide biological insights that would otherwise go undetected (Mooney and Wilmot, 2015; Tragante et al., 2017).

#### 2.3.3 Annotation coverage

Another important characteristic to carefully consider is the database’s genome and gene coverage (i.e., the percentage of the genome covered and the number of terms per gene, respectively). Resources with a limited number of genes and functional terms, or with biases towards certain genes, may lead to undetected biological descriptions in FEA, possibly hindering the biological relevance of its results (Haynes et al., 2018; Gable et al., 2022). Databases with lower coverage, such as KEGG pathways and HPO, have been shown to significantly affect ORA methods (Ziemann et al., 2024). Such effects may be species-dependent since annotation coverage can vary across organisms within the same database (Gable et al., 2022). Hence, comprehensive databases, such as the GO, are generally recommended to be coupled with targeted databases, which are expected to exhibit lower annotation coverage (Mooney and Wilmot, 2015; Ziemann et al., 2024).

#### 2.3.4 Annotation system version

To incorporate newly generated knowledge, functional databases are frequently updated, changing the biological information. As a result, the use of different versions of annotation systems in FEA can yield different results, potentially even altering the biological interpretation of the same input (Tomczak et al., 2018). However, most FEA tools that embed such resources use different update schedules, which may lead to the unintentional use of outdated annotation versions. In this sense, users should be aware of the database version used by the selected tool and use the most up-to-date releases whenever possible to manually input annotations. Additionally, it is essential that researchers report the exact versions used in their analyses to ensure reproducibility (Tomczak et al., 2018; Wijesooriya et al., 2022).

### 2.4 How to effectively extract biological insights

FEA usually outputs a table containing enriched terms and relevant information columns. The information provided varies across platforms, but it should generally include, for each term, the statistical significance (both raw and corrected values), the term size, the detected input genes, and a fold enrichment metric. These statistics are crucial for extracting the most relevant enriched terms, since FEA can produce extremely large results that are difficult to interpret. In this context, because the last step of FEA inherently involves manual interpretation, post-processing and visualization techniques are essential to enable the biological interpretation of the results.

When handling large results, an efficient initial strategy is to filter out non-statistically significant results and, if applicable, order the enriched terms by corrected p-values or fold enrichment. Additionally, it is possible to remove less informative terms while maintaining the analysis’s biological relevance. Typically, larger terms are associated with broader generic biological descriptions. Gable et al. (2022) found that terms encompassing between 100 and 200 genes provide the ideal balance between detectability (i.e., the probability of enriching a given term) and biological specificity. Thus, removing terms outside this range may help researchers reduce noise and improve interpretability.

In addition to simple filtering strategies, numerous approaches leverage the information in FEA results to facilitate interpretation. Various redundancy-removal techniques that exploit the functional data in annotation databases to eliminate overlapping descriptions have been developed. However, users should be cautious when employing such strategies, as the smaller, more informative terms, which tend to have less statistical power (Gable et al., 2022), are often removed. For instance, the *orsum* (Ozisik et al., 2022) library in Python uses relationships among pathways in hierarchically organized databases to remove redundant terms while still maintaining some biological specificity when a more specific term is statistically more significant than its ancestor. An alternative to this is clustering algorithms that group functionally related terms, providing an overview of the broad biological processes yielded in the FEA. Moreover, this also enables specific visualizations that further facilitate the interpretation of the resulting biological significance. ViSEAGO (Brionne et al., 2019) employs hierarchical clustering to group and rank FEA results, generating heatmaps and MDS plots that highlight the most relevant biological descriptions yielded. However, most visualization tools support only one or a few databases (most commonly GO, Reactome, and KEGG), thus making it necessary to develop tools that can handle any annotation system. The GeneSetCluster R package (Ewing et al., 2020) harmonizes functional terms based on their respective gene sets, eliminating the need to use structural database information and facilitating comparisons across results from different tools.

Given the importance of FEA and the challenge of interpreting its results, a plethora of tools for FEA interpretation have been developed in recent years to support users. For a thorough review of the state of the art in interpretation strategies and tools, we direct readers to (Ryan et al., 2025) for a comprehensive assessment of these approaches.

### 2.5 Artificial intelligence and machine learning in FEA tools

In recent years, AI has been increasingly used as a resource to enhance the effectiveness of FEA. Although AI is not a statistical replacement for FEA methods, AI-based strategies are being implemented in FEA to support the analysis.

For instance, to assist in extracting biological meaning from FEA, EnrichGT (Wang et al., 2026) employs an LLM to label clusters of functionally related enriched descriptions and generate a short background text on the depicted biological theme, thereby facilitating human interpretation.

Moreover, AI has recently been employed to generate gene set functional descriptions, enabling users to circumvent limited annotation coverage and identify novel functions not yet annotated in functional databases (Hu et al., 2025; Wang et al., 2025). However, it is important to clarify that such resources do not replace FEA, as they do not perform any enrichment statistical analysis; rather, they provide functional insights into gene lists that can support FEA. Also, these tools are prone to hallucinations, even though they might have self-verification systems, such as GeneAgent (Wang et al., 2025).

Recently, tools that incorporate AI strategies within their FEA pipelines have been developed primarily to combine results from different methods. KOBAS-i (Bu et al., 2021) implements a machine learning approach that leverages multiple FEA methods to compute a relevance score for each enriched term yielded to rank the results. Similarly, the recent PAG-Agent (Nguyen et al., 2026) provides an AI agent-assisted FEA workflow that supports the user throughout the whole process. The agent can run multiple FEA methods and generate consensus results that are ranked according to their relevance to the biological context of the experiment informed by the user. PAG-Agent also generates visualization plots and allows the user to interact with the model via chat to support interpretation of results.

In addition, recent resources have begun incorporating ML approaches into the core of their FEA strategies. For example, GNNEnrich (Mziou-Sallami et al., 2025) uses a graph neural network to generate protein embeddings. The tool leverages the correlation between query gene embeddings and genes in functional terms to calculate enrichment scores, which are then compared against random queries’ scores to determine significance.

While AI is still in its infancy in the field of FEA, it may provide rather useful resources to users by facilitating interpretation, bypassing limitations or simply increasing the efficiency of the analysis. Nonetheless, efforts still need to be made to validate such tools and conscientiously define its limitations.

### 2.6 Guidelines in a nutshell

#### 2.6.1 Define the biological question before selecting a tool

FEA should be guided by the biological question rather than performed as an automatic step. Exploratory studies aimed at identifying broad biological themes may benefit from tools that highlight dominant, widely annotated processes. Functional, hypothesis-oriented studies seeking pathway-level insight may require tools that prioritize more specific terms or apply stricter filtering.

#### 2.6.2 Carefully construct the input data

When conducting FEA with FCS, PT, or NB tools that take expression matrices as input (e.g., GSEA, NetGSA, and CAMERA), researchers should ensure that the data have been carefully preprocessed through normalization and imputation of missing values. If employing ORA, users should carefully consider logFC and p-value thresholds, as well as the stringency of the statistical test, when selecting genes, and ensure that the list reflects the underlying biological question. Input data that was not correctly constructed (as guided by the research question) will lead to misguided and misinterpreted results that might be confused by technical artifacts.

#### 2.6.3 Combine different functional databases

Functional databases contain different focuses and structural characteristics. Being aware of the used database’s features may help researchers evidence the biological knowledge yielded. Additionally, combining resources with different knowledge bases, such as using HPO (a disease phenotype database) alongside Reactome (primarily a pathway database), may help researchers identify hidden associations and complement the interpretation of results. Relying on a single database might lead to being solely led by a single perspective in a world of tools with distinct methodologies.

#### 2.6.4 Combine tools of different method classes

Each FEA class incorporates varying types of biological information. Leveraging these differences by employing distinct methods can help researchers validate findings and identify terms that are detected exclusively by a given class. Platforms that support multiple methods, such as WebGestalt, clusterProfiler, and CePa (Table 1), are recommended for this purpose because they facilitate the integration of results.

#### 2.6.5 Understand that enrichment tools are not necessarily compatible

Different enrichment platforms implement distinct annotation management, background definitions, ranking strategies, and filtering approaches. These methodological differences can lead to substantial variation in the number, ranking, and specificity of enriched terms. Evaluate which tools are the best for your research goals. There are no shortcuts to this step; each research question demands a focused mindset on that given biological issue.

#### 2.6.6 Interpret enrichment results beyond p-values

Statistical significance alone does not guarantee biological relevance. Researchers should consider GO specificity, biological coherence, and consistency with known mechanisms when interpreting enrichment outputs. Relying solely on p-values might lead to oversimplifications or to extracting conclusions based on generic terms that may have the most significant p-values, even though they are not necessarily the ones that best explain the research question.

#### 2.6.7 Manage redundancy among enriched terms

FEA often produces overlapping or semantically similar terms. Clustering, semantic similarity filtering, or manual curation can help identify the most informative biological themes. Automation might be an enemy in this step.

#### 2.6.8 Employ effective visualization strategies

FEA can produce massive results that may be difficult to interpret. In such cases, visualization strategies, such as heatmaps, tree maps, pathway interaction networks, and term relationship graphs, substantially facilitate biological interpretation. Users should prioritize FEA tools that implement these functions (e.g., clusterProfiler, g:Profiler, and WebGestalt) or employ specific FEA visualization tools, such as orsum, ViSEAGO, and GeneSetCluster.

## 3 Discussion

FEA is vital to the interpretation of gene lists commonly generated by OMICS experiments, since it translates them into biological functions strongly associated with them. However, the field is far from unified, making it hard for users to find practical information in the literature and leading to a rich-gets-richer effect, in which the most popular tools and databases are used carelessly despite methodological advancements. In these guidelines, we addressed the issue by extracting actionable insights from multiple sources and providing tangible information on the strategies underpinning FEA for newcomers.

The decision process behind FEA begins with the user’s biological question of interest. For explorative purposes, with the aim of uncovering novel gene associations with phenotypes, tools that account for gene expression and interaction data (i.e., FCS, PT, and NB methods) are ideal. For validating expected associations, conservative approaches, such as ORA-based methods and CAMERA, are suitable as they tend to yield fewer false positives (Buzzao et al., 2024). The input data also plays a role in this choice: for simple gene lists without expression values, ORA and NB tools are applicable; for gene lists with experimental information or whole expression matrices, FCS, PT, and NB are preferred. Moreover, combining different methods enhances FEA by enabling researchers to validate strong associations and potentially extend the scope of enriched descriptions. Nevertheless, such recommendations rely mainly on the methodological strategy itself and on the few, non-standardized performance studies that often find conflicting results (Xie et al., 2021). The FEA field lacks an established gold standard for comparing tools, making it extremely difficult to confidently select the best-performing approaches. In this regard, addressing this limitation must become a central priority for the FEA field moving forward.

The biological question also guides the selection of the annotation system. Each database can contain different types of biological information; therefore, using descriptions that suit the user’s goals and combining them strengthens the hypothesis in question. General database recommendations include using up-to-date, comprehensive, hierarchically structured resources (e.g., GO and Reactome), which perform better in enrichment analyses (Gable et al., 2022; Ziemann et al., 2024). However, very few studies have systematically assessed functional databases with respect to their behavior in FEA. New efforts are needed to develop comprehensive, practical guidance in this department.

While FEA is in itself an interpretation strategy, it often requires additional techniques to facilitate the biological interpretation of the results. For this end, removing broad, redundant terms, clustering related descriptions, and employing graphical visualizations are among the most effective strategies. Most enrichment platforms contain built-in features that facilitate interpretation (Table 1). However, we find that such implementations are often limited; thus, specialized FEA visualization tools, such as orsum, ViSEAGO, and GeneSetCluster, are recommended for users seeking alternatives.

The FEA is most definitely not the straightforward procedure researchers commonly think it is. Its success relies significantly on the users’ choices, which are numerous throughout the whole analysis. The general practical guide provided in these guidelines helps users ask the right questions and make the appropriate choices to make the most out of FEA.

## Data Availability

The original contributions presented in the study are included in the article/supplementary material, further inquiries can be directed to the corresponding author.
